# Supplementary material for: Transferability of Cardiopulmonary Parameters between Treadmill and Cycle Ergometer Testing in Male Triathletes—Prediction Formulae
Source: Int J Environ Res Public Health. 2022 Feb 6;19(3):1830. doi: 10.3390/ijerph19031830 (PMC8834943; doi:10.3390/ijerph19031830)
Supplement: Supplementary file 1 [file ijerph-19-01830-s001.zip › Supplementary Table S1. CPET characteristics.pdf]

Supplementary Table S1. CPET characteristics.

CPET characteristics								
	TE				CE			
**Variable**	**Mean**	**CI**		**SD**	**Mean**	**CI**		**SD**
		-95%	95%			-95%	95%	
VO_{2AT}	38.30	37.56	39.04	4.62	32.52	31.67	33.36	5.27
VO_{2ATA}	2970.25	2913.35	3027.15	355.05	2512.01	2455.11	2568.91	355.05
RER_{AT}	0.89	0.88	0.89	0.04	0.86	0.85	0.87	0.05
HR_{AT}	147.59	145.90	149.27	10.52	134.38	132.47	136.28	11.89
VE_{AT}	81.86	80.10	83.63	11.01	65.63	63.96	67.29	10.39
fR_{AT}	36.54	35.38	37.71	7.27	28.50	27.70	29.30	5.00
Lac_{AT}	1.80	1.73	1.87	0.44	1.70	1.64	1.76	0.38
VO_{2RCP}	47.28	46.35	48.20	5.77	43.55	42.36	44.74	7.43
VO_{2RCPA}	3665.26	3595.82	3734.70	433.30	3369.25	3289.07	3449.42	500.28
VO_{2RCP}	3698.03	3622.19	3773.88	421.37	3421.56	3346.81	3496.31	415.31
RER_{RCP}	1.00	1.00	1.00	0.03	1.01	1.00	1.01	0.02
HR_{RCP}	170.90	169.27	172.53	10.19	161.54	159.71	163.37	11.44
VE_{RCP}	118.22	115.68	120.77	15.89	109.08	106.24	111.92	17.72
fR_{RCP}	45.37	43.91	46.82	9.09	38.90	37.76	40.05	7.16
Lac_{RCP}	4.22	4.10	4.34	0.74	4.23	4.14	4.33	0.62
VO_{2max}	51.73	50.74	52.72	6.20	49.15	48.04	50.26	6.93
VO_{2maxA}	4008.06	3936.89	4079.23	444.10	3799.83	3725.04	3874.62	466.70
RER_{max}	1.11	1.10	1.11	0.03	1.09	1.07	1.11	0.14
HR_{max}	181.91	180.25	183.57	10.36	176.18	174.51	177.86	10.44
VE_{max}	150.64	147.60	153.69	19.01	157.42	153.45	161.38	24.74
fR_{max}	58.10	56.55	59.65	9.67	56.58	55.05	58.12	9.58
Lac_{max}	9.52	9.25	9.80	1.71	12.11	10.42	13.80	10.50
S_{AT}/P_{AT}*	11.20	10.97	11.43	1.43	177.83	172.91	182.76	30.73
S_{RCP}/P_{RCP}*	14.28	14.01	14.55	1.69	260.77	254.40	267.13	39.73
S_{max}/P_{max}*	16.15	15.86	16.43	1.76	311.06	303.86	318.27	44.96

Footnote: Abbreviations: CPET, cardiopulmonary exercise test; TE, trademill; CE, cycle ergonometer; CI, confidence interval; SD, standard deviation; VO_{2AT}, relative VO_2 at AT ($mL \cdot min^{-1} \cdot kg^{-1}$); VO_{2ATA}, absolute VO_2 at AT ($mL \cdot min^{-1}$); RER_{AT}, respiratory exchange ratio at AT; HR_{AT}, heart rate at AT (bpm); VE_{AT}, pulmonary ventilation at AT ($L \cdot min^{-1}$); fR_{AT}, respiratory frequency at AT (breaths per minute); Lac_{AT}, lactate concentration at AT ($mmol \cdot L^{-1}$); VO_{2RCP}, relative VO_2 at RCP ($mL \cdot min^{-1} \cdot kg^{-1}$); VO_{2RCPA}, absolute VO_2 at RCP ($mL \cdot min^{-1}$); VO_{2RCP}, relative VO_2 at RCP ($mL \cdot min^{-1} \cdot kg^{-1}$); RER_{RCP}, respiratory exchange ratio at RCP; HR_{RCP}, heart rate at RCP (bpm); VE_{RCP}, pulmonary ventilation at RCP ($L \cdot min^{-1}$); fR_{RCP}, respiratory frequency at RCP (breaths per minute); Lac_{max}, lactate concentration at RCP ($mmol \cdot L^{-1}$); VO_{2max}, relative maximal VO_2 ($mL \cdot min^{-1} \cdot kg^{-1}$); VO_{2maxA}, absolute maximal VO_2 ($mL \cdot min^{-1}$); RER_{max}, maximal

respiratory exchange ratio; HR_{max}, maximal heart rate (bpm); VE_{max}, maximal pulmonary ventilation (L·min^{-1}); fR_{max}, maximal respiratory frequency; Lac_{max}, maximal lactate concentration (mmol·L^{-1}); S_{AT}, speed at AT (km·h^{-1}); P_{AT}, power at AT (watt); S_{RCP}, speed at RCP (km·h^{-1}); P_{RCP}, power at RCP (watt); S_{max}, maximal speed (km·h^{-1}); P_{max}, maximal power (watt).
*speed for TE, power for CE.
