# Supplementary material for: Transferability of Cardiopulmonary Parameters between Treadmill and Cycle Ergometer Testing in Male Triathletes—Prediction Formulae
Source: Int J Environ Res Public Health. 2022 Feb 6;19(3):1830. doi: 10.3390/ijerph19031830 (PMC8834943; doi:10.3390/ijerph19031830)
Supplement: Supplementary file 1 [file ijerph-19-01830-s001.zip › Supplementary Table S2. Additional cycling prediction equations (1).pdf]

Supplementary Table S2. Additional cycling prediction equations.

Category	Multiple Regression Equation	MAE	Adjusted R^2
**VO_{2AT}** *(eqs)*	$10.65_{[5.37]SE} + 0.55_{[0.12]SE} x\ RVO_{2AT} - 0.06_{[0.03]SE} x\ RVE_{AT} - 0.07_{[0.03]SE} x\ RHR_{AT} - 0.36_{[0.17]SE} x\ RLac_{max} + 0.39_{[0.08]SE} x\ RVO_{2max}$	2.26	0.70
**VO_{2AT}** *(eqms)*	$-7.20_{[2.69]SE} + 0.33_{[0.10]SE} x\ RVO_{2AT} + 0.53_{[0.07]SE} x\ RVO_{2max}$	2.36	0.66
**HR_{AT}** *(eqs)*	$2.48_{[14.26]SE} + 0.40_{[0.12]SE} x\ RHR_{AT} + 0.40_{[0.12]SE} x\ RHR_{max}$	6.52	0.45
**HR_{AT}** *(eqms)*	– – –	– – –	– – –
**VO_{2RCP}** *(eqs)*	$-1.73_{[5.79]SE} + 0.80_{[0.14]SE} x\ RVO_{2AT} - 0.004_{[0.001]SE} x\ RVO_{2ATA} - 0.56_{[0.18]SE} x\ RLac_{max} + 0.46_{[0.08]SE} x\ RVO_{2max} + 0.64_{[0.24]SE} x\ BMI - 0.40_{[0.11]SE} x\ BF$	2.49	0.77
**VO_{2RCP}** *(eqms)*	$5.53_{[4.14]SE} + 0.64_{[0.14]SE} x\ RVO_{2AT} - 0.003_{[0.001]SE} x\ RVO_{2ATA} + 0.49_{[0.09]SE} x\ RVO_{2max} - 0.25_{[0.10]SE} x\ BF$	2.65	0.74
**HR_{RCP}** *(eqs)*	$18.94_{[10.80]SE} + 0.82_{[0.06]SE} x\ RHR_{max} - 0.36_{[0.14]SE} x\ BF$	4.85	0.64
**HR_{RCP}** *(eqms)*	$12.43_{[10.77]SE} + 0.82_{[0.06]SE} x\ RHR_{max}$	4.88	0.62
**VO_{2max}** *(eqs)*	$6.75_{[4.06]SE} + 0.07_{[0.03]SE} x\ RfR_{RCP} + 0.51_{[0.12]SE} x\ RVO_{2max} + 0.40_{[0.13]SE} x\ RVO_{2RCP} - 0.39_{[0.09]SE} x\ BF$	2.59	0.76
**VO_{2max}** *(eqms)*	$12.05_{[4.06]SE} + 0.84_{[0.06]SE} x\ RVO_{2max} - 0.41_{[0.09]SE} x\ BF$	2.80	0.75
**HR_{max}** *(eqs)*	$25.04_{[8.10]SE} + 0.25_{[0.13]SE} x\ RHR_{RCP} + 0.61_{[0.27]SE} x\ RLac_{max} + 0.59_{[0.12]SE} x\ RHR_{max} - 0.32_{[0.10]SE} x\ BF$	3.33	0.77
**HR_{max}** *(eqms)*	$20.36_{[8.25]SE} + 0.87_{[0.05]SE} x\ RHR_{max}$	3.61	0.75

Abbreviations: MAE, mean absolute error; VO2AT, relative VO2 at AT (mL·min-1·kg-1); eqs, eqation in the simplified form; RVO2AT, running relative VO2 at AT (mL·min-1·kg-1); RVEAT, running pulmonary ventilation at AT (L·min-1); RHRAT, running heart rate at AT (bpm); RLacmax, running maximal lactate concentration (mmol·L-1); RVO2max, running relative maximum VO2 (mL·min-1·kg-1); eqms, equation in the most simplified form; RHRmax, running maximal heart rate (bpm); RVO2ATA, running absolute VO2 at AT (mL·min-1); BMI, body mass index (kg·m-2); BF, body fat (%); RfRRCP, running respiratory frequency at RCP (breaths per minute); RVO2RCP, running relative VO2 at RCP (mL·min-1·kg-1); RHRRCP, running heart rate at RCP (bpm).
