# Supplementary material for: Transferability of Cardiopulmonary Parameters between Treadmill and Cycle Ergometer Testing in Male Triathletes—Prediction Formulae
Source: Int J Environ Res Public Health. 2022 Feb 6;19(3):1830. doi: 10.3390/ijerph19031830 (PMC8834943; doi:10.3390/ijerph19031830)
Supplement: Supplementary file 1 [file ijerph-19-01830-s001.zip › Supplementary Table S3. Additional running prediction equations.pdf]

Supplementary Table S3. Additional running prediction equations.

Category	Multiple Regression Equation	MAE	Adjusted R^2
**VO$_{2AT}$** *(eqs)*	$17.03_{[0.176]SE} + 0.49_{[0.04]SE} \times cVO_{2RCP}$	2.21	0.56
**VO$_{2AT}$** *(eqms)*	– – –	– – –	– – –
**HR$_{AT}$** *(eqs)*	$43.84_{[17.50]SE} - 0.76_{[0.36]SE} \times cVO_{2AT} - 0.002_{[0.08]SE} \times cVE_{AT} + 0.74_{[0.07]SE} \times cHR_{max} + 0.39_{[0.27]SE} \times cVO_{2RCP} - 1.20_{[0.50]SE} \times BMI + 0.73_{[0.25]SE} \times BF$	5.36	0.50
**HR$_{AT}$** *(eqms)*	$8.06_{[12.89]SE} + 0.76_{[0.07]SE} \times cHR_{max} + 0.42_{[0.16]SE} \times BF$	5.72	0.50
**VO$_{2RCP}$** *(eqs)*	$14.67_{[2.57]SE} + 0.23_{[0.12]SE} \times cVO_{2AT} - 0.08_{[0.36]SE} \times cVE_{AT} + 0.08_{[0.06]SE} \times cfR_{AT} + 0.04_{[0.01]SE} \times cP_{AT} - 0.45_{[0.09]SE} \times cVO_{2max}$	2.44	0.72
**VO$_{2RCP}$** *(eqms)*	$12.83_{[2.11]SE} + 0.60_{[0.04]SE} \times cVO_{2max} + 0.03_{[0.01]SE} \times cP_{AT}$	2.28	0.71
**HR$_{RCP}$** *(eqs)*	$67.13_{[13.00]SE} - 1.17_{[0.31]SE} \times cVO_{2AT} - 0.07_{[0.02]SE} \times cVE_{max} + 0.01_{[0.002]SE} \times cVO_{2ATA} + 0.77_{[0.05]SE} \times cHR_{max} + 0.49_{[0.21]SE} \times cVO_{2RCP} - 0.15_{[0.05]SE} \times Age - 0.59_{[0.28]SE} \times BMI$	3.47	0.76
**HR$_{RCP}$** *(eqms)*	$33.70_{[8.69]SE} - 0.25_{[0.09]SE} \times cVO_{2AT} + 0.83_{[0.05]SE} \times cHR_{max}$	3.80	0.71
**VO$_{2max}$** *(eqs)*	$28.49_{[5.23]SE} + 0.39_{[0.15]SE} \times cVO_{2AT} - 0.06_{[0.03]SE} \times cHR_{AT} - 0.21_{[0.08]SE} \times cfR_{AT} - 0.003_{[0.001]} \times cVO_{2ATA} - 0.03_{[0.01]} \times cP_{AT} - 0.14_{[0.06]} \times cfR_{RCP} + 0.49_{[0.09]SE} \times cVO_{2max} - 0.07_{[0.03]SE} \times Age$	2.38	0.74
**VO$_{2max}$** *(eqms)*	$16.51_{[2.10]SE} + 0.26_{[0.12]SE} \times cVO_{2AT} + 0.55_{[0.09]SE} \times cVO_{2max}$	2.57	0.71
**HR$_{max}$** *(eqs)*	$89.45_{[26.65]SE} - 0.25_{[0.09]SE} \times cVO_{2AT} - 0.02_{[0.02]SE} \times cVE_{max} - 28.49_{[24.10]SE} \times cRER_{RCP} + 0.84_{[0.05]SE} \times cHR_{max} - 0.14_{[0.05]SE} \times Age - 0.60_{[0.30]SE} \times BMI + 0.30_{[0.16]SE} \times BF$	3.35	0.79
**HR$_{max}$** *(eqms)*	$27.43_{[8.18]SE} + 0.88_{[0.05]SE} \times cHR_{max}$	3.72	0.75

Abbreviations: MAE, mean absolute error; VO2AT, relative VO2 at AT (mL·min-1·kg-1); CVO2RCP, cycling relative VO2 at RCP (mL·min-1·kg-1); eqs, equation in the simplified form; eqms, equation in the most simplified form; HRAT, heart rate at AT (bpm); CVO2AT, cycling relative VO2 at AT (mL·min-1·kg-1); CVEAT, cycling pulmonary ventilation at AT (L·min-1); CHRmax, cycling maximal heart rate (bpm); BMI, body mass index (kg·m-2); BF, body fat (%); VO2RCP, relative VO2 at RCP (mL·min-1·kg-1); CfRAT, cycling respiratory rate at AT (breaths per minute); CPAT, cycling power at AT (watt); CVO2max, cycling relative maximum VO2 (mL·min-1·kg-1); HRRCP, heart rate at RCP (bpm); CVEmax, cycling maximal pulmonary ventilation (L·min-1); CVO2ATA, cycling absolute VO2 at AT (mL·min-1); Age, age (years); VO2max, relative maximum VO2 (mL·min-1·kg-1); CHRAT, cycling heart rate at AT (bpm); CfRRCP, cycling respiratory rate at RCP (breaths per minute); HRmax, maximal heart rate (bpm); CRERRCP, cycling respiratory exchange ratio at RCP.
